# Descriptive Kinematic Analysis of the Potentially Tragic Accident at the 2020 Austrian MotoGP Grand Prix Using Low-Cost Instruments: A Brief Report

**DOI:** 10.3390/ijerph17217989

**Published:** 2020-10-30

**Authors:** Marco Gervasi, Erica Gobbi, Valentina Natalucci, Stefano Amatori, Fabrizio Perroni

**Affiliations:** Department of Biomolecular Sciences, University of Urbino, 61029 Urbino, Italy; erica.gobbi@uniurb.it (E.G.); valentina.natalucci@uniurb.it (V.N.); s.amatori1@campus.uniurb.it (S.A.); fabrizio.perroni@uniurb.it (F.P.)

**Keywords:** MotoGP, video analysis, collision, accident, safety

## Abstract

Background: During the first Austrian MotoGP Grand Prix of 2020, following a serious accident involving the riders J. Zarco and F. Morbidelli, Morbidelli’s riderless bike cartwheeled across turn 3, narrowly missing V. Rossi and M. Viñales by just a few centimeters. As is the case with ordinary traffic accidents, analyzing the dynamics of motorcycle racing accidents can help improve safety; however, to date, the literature lacks studies that analyze the causes and severity of such accidents. Hence, the purpose of this study was to analyze the main causes that led to the accident at the 2020 Austrian MotoGp Grand Prix, to quantify the speeds and distances of the bikes and riders involved, and to hypothesize several alternative scenarios using a low-cost method. Method: Kinovea and Google Earth Pro software were used to identify markers along the racetrack and to measure the distances and calculate the time it took the motorcycles to cover those distances. The analyses were carried out on three 30-fps (frames per second) videos. Results: Zarco’s average speed as he was overtaking Morbidelli on the straightaway before turn 2 was 302 ± 1.8 km/h, higher than that of Rins and Rossi (299.7 ± 1.7 and 296 ± 1.7 km/h, respectively). The speed of Zarco and Rossi’s bikes 44.5 m before the crash was the same (267 ± 7.9 km/h). Immediately after overtaking Morbidelli, Zarco moved 2.92 m towards the center of the racetrack from point A to B, crossing Morbidelli’s trajectory and triggering the accident. Morbidelli’s riderless bike flew across turn 3 at a speed of about 76 km/h, missing V. Rossi by just 20 cm. The consequences could have been catastrophic if Rossi had not braked just 0.42 s before encountering Morbidelli’s bike in turn 3. Conclusion: Through a low-cost quali-quantitative analysis, the present study helps us to gain a deeper understanding of the dynamics of the accident and its main causes. Furthermore, in light of our findings regarding the dynamics and severity of the accident and the particular layout of the Red Bull Ring circuit, racers should be aware that overtaking at the end of turn 2, following the same trajectory as the riders involved in the crash, could be very risky.

## 1. Introduction

Motorcycle racing in the elite MotoGP class attracts millions of spectators worldwide, with riders competing in 18 races across the globe each year. Crashes are a rare but regular feature of elite motorcycle racing [1,2,3], with a prevalence of 9.7 per hundred rider hours, while injuries are uncommon, occurring in only 9% of crashes [4]. In a recent study, Bedolla et al. identified four basic crash types in elite motorcycle racing: lowside, highside, topside, and collision crashes [4]. The causes and dynamics of such accidents are multiple and, without reliable data, difficult to understand. However, as is often done in the case of ordinary traffic accidents, a reliable analysis of motorcycle crash dynamics can be made using video footage, providing us with insights into the causes of the accident and, in some cases, leading to improvements in safety conditions [5,6,7,8].

**Figure 1:**
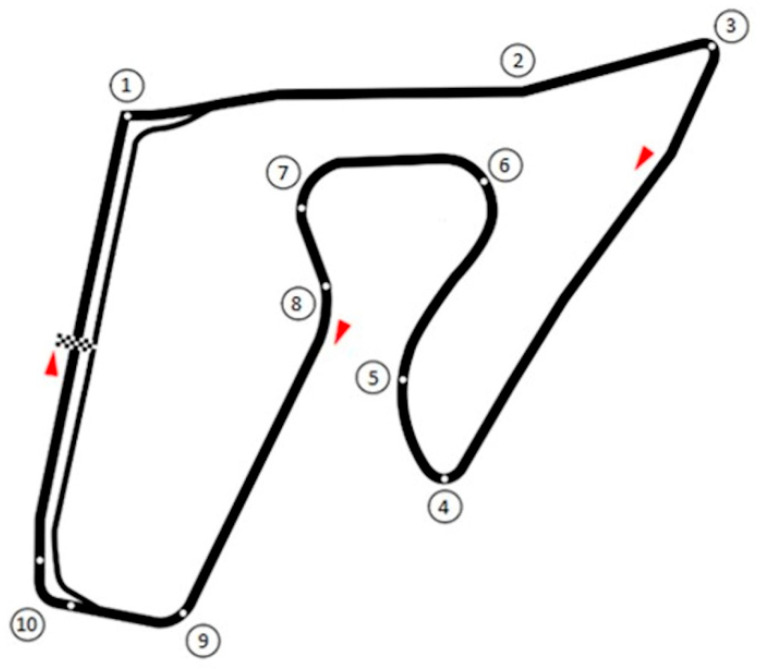
An overview of the Red Bull Ring circuit, Austria: points from 1 to 10 represent the turns in the track.

In particular, in the case of ordinary traffic accidents, the video footage that is analyzed comes from safety cameras installed in vehicles, from smartphones [5,8], or from surveillance cameras found in the area of the accident [7]. This footage is then analyzed using software that allows us to precisely measure distances, speed, and angles and hence, reconstruct specific trajectories. Nevertheless, in these cases, there are limitations stemming from the difficulty of obtaining video footage from different angles, as well as limitations related to the frequency of the frames per second of the images, which can vary according to the type of camera being used. On the contrary, elite MotoGp motorcycle races are filmed with cameras providing top quality images from multiple angles, making video analyses based on such footage more precise. Unfortunately, in the literature, there are few studies investigating the dynamics of accidents in motorcycle racing [6]. This lack of research is probably due to the inherent difficulty of obtaining data even though such sporting events are monitored by high definition cameras and multiple high precision sensors. In the absence of this sort of high-quality data, the analysis of television footage using low-cost instruments can provide insights into the dynamics and seriousness of accidents, as well as other key factors contributing to accidents, to improve race safety conditions. In this brief report, we analyzed the potentially catastrophic accident that occurred at the MotoGP Grand Prix in Austria on 16 August 2020. Veteran rider Valentino Rossi and his teammate Maverik Viñales miraculously emerged from the crash unscathed, narrowly avoiding being struck by not just one, but two airborne motorbikes. The circuit at the Austrian Grand Prix, namely the “Red Bull Ring” (see Figure 1), is among the fastest in the world [9], featuring numerous stretches where riders reach speeds of over 300 km/h, followed by sections that require them to slow down to 50–60 km/h. The total length of the track is 4318 m and it features 10 turns with a vertical drop of 65 m. In the specific case of the 2020 Austrian Grand Prix, the contact that occurred between the two bikes could easily have resulted in an unprecedented tragedy.

### Accident Description

It all unfolded on the eighth lap of the race, as the riders raced down the straightaway between turns 2 and 3 (see Video S1). Until the moment of the crash, the riders were in the following order, from first to tenth: P. Espargaró, A. Dovizioso, J. Miller, J Mir, M. Oliveira, M. Viñales, V. Rossi, F. Morbidelli, J. Zarco, and A. Rins. The accident occurred after turn 2. Zarco had just overtaken Morbidelli and was preparing to enter turn 3, but moments after overtaking the other rider, Zarco’s rear wheel came into contact with the front wheel of Morbidelli’s bike, setting off a terrible chain of events. Following the contact, the two riders were thrown from their bikes, which continued careening down the track at high speed along different trajectories. Morbidelli’s bike proceeded along the outer edge of the track in proximity to the curb, crossing over turn 3 and maintaining the same trajectory that it had been on in the straightaway leading into the curve. Zarco’s bike followed a different trajectory, crashing into barriers, which, nonetheless, did not prevent it from careening across turn 3 an instant before Morbidelli’s bike. Viñales and Rossi were rounding turn 3 just as the two bikes cartwheeled across their paths. Miraculously, Morbidelli and Zarco’s bikes missed the riders by only a few centimeters. Zarco’s bike, after striking the barriers, flew across the curve just a few centimeters over Vinales’ helmet, while Morbidelli’s bike sliced between Viñales (in front) and Rossi, crossing the latter rider’s path. This accident has been the focus of a great deal of media attention, and in its wake, there have been numerous unconfirmed reports regarding the factors that may have led to the crash. Hence, the aim of the present study was to perform an analysis of the available race footage to answer the following questions: What were the main factors and dynamics that led to the accident? How fast was Morbidelli’s bike travelling as it careened across Rossi’s path? How close did Morbidelli’s bike come to striking Rossi and why did it miss him? Can we quantify the hypothetical impact of Morbidelli’s bike with Valentino Rossi?

## 2. Materials and Methods

**Figure 2:**
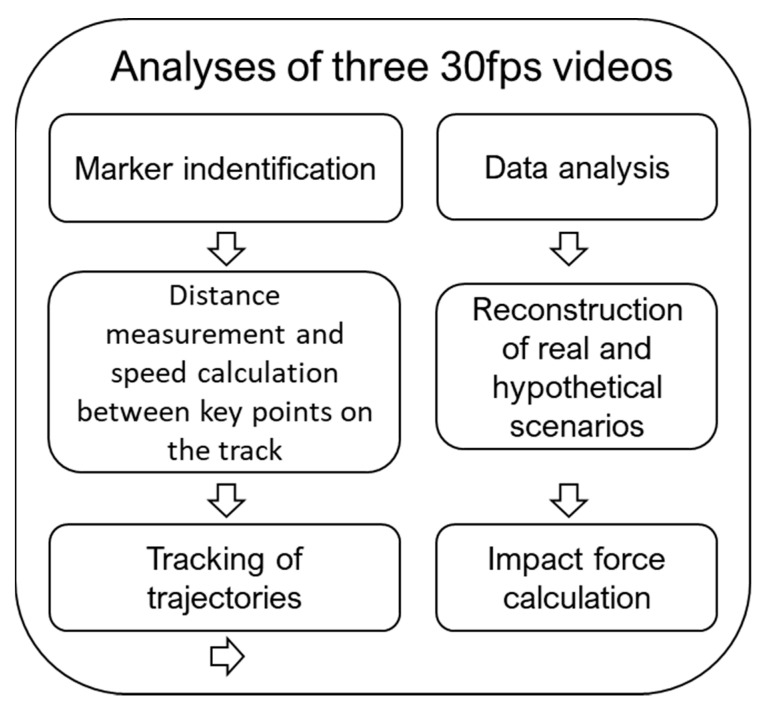
Chart showing the analytical procedures applied in the study.

The analysis of the available race footage was developed according the procedure showed in Figure 2.

**Figure 3:**
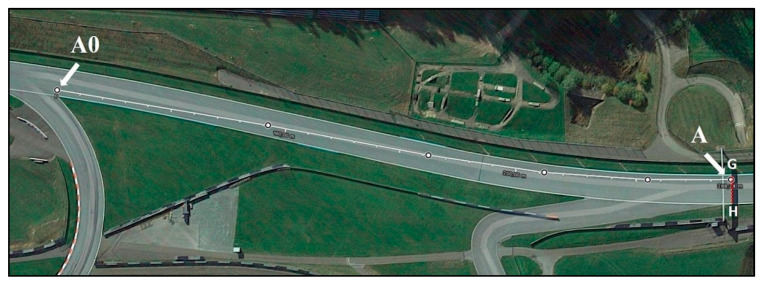
Satellite image showing the section of the straightaway from point A0 (corresponding to the point where the asphalt ends and the grass begins on the outer edge of the track) to point A (line between points G and H, where a shadow was cast directly under the “myWorld” billboard). The distance from A0 to A measures 288 m.

**Figure 4:**
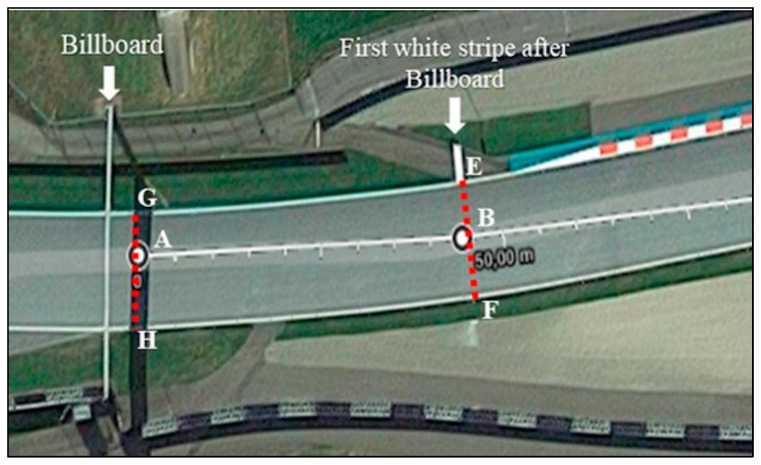
Satellite image showing the section of the straightaway before turn 3. The white arrows indicate a billboard and the first white stripe on the left side of the track (race direction). The red dashed lines were traced from point G to point H and from point E to point F. Points A and B indicate the crossing of the front wheel of Zarco’s bike, as well as the front wheels of the other riders under consideration. The distance from point A to point B measures 44.5 m.

**Figure 5:**
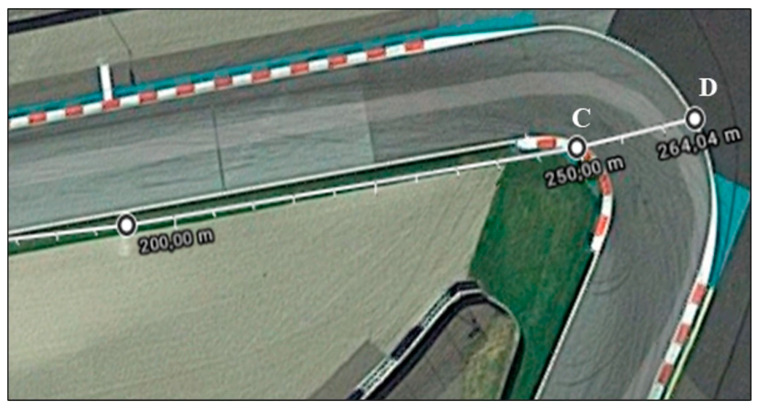
Satellite image showing the section of the straightaway leading into turn 3. Point C corresponds to a marker on the internal curb of turn 3, the point where Morbidelli’s bike began cartwheeling across the track. Point D is shown by a marker on the outer edge of turn 3 with a trajectory that follows the line of the straightaway, the same trajectory that Morbidelli’s bike followed as it cartwheeled across the curve. The line from C to D measures 14 m.

The analysis was performed using Kinovea software (Kinovea ver. 0.8.15, open source project) [10] applied to three 30 fps (frames per second) videos (30 fps means that the camera captured 30 frames in a single second of video). Due to the video frame rate that limits precision to 0.03 s, we incorporated this error into the calculated speeds, showing the upper and lower average error estimates. Google Earth Pro software (Google LLC, Mountain View CA, USA) was used to measure the distance between the key points on the racetrack (see Figure 3, Figure 4 and Figure 5). This software is scientifically validated for measurements of this kind [11]. Finally, Microsoft Excel (Microsoft Corporation, Redmond, WA, USA) was used to perform data analysis and to create the figures.

**Figure 6:**
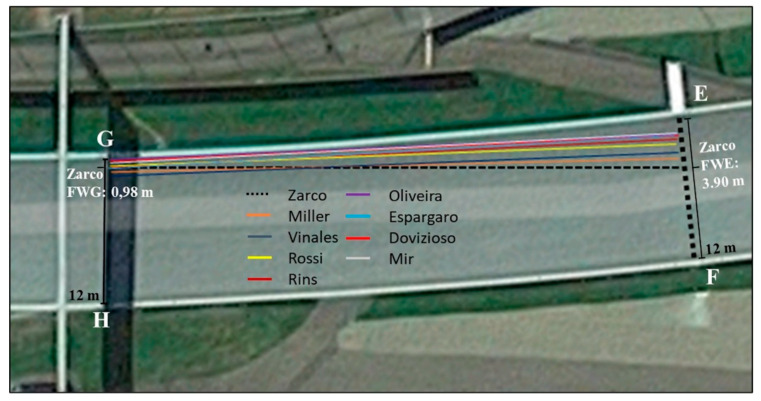
Distance between point G and point H: 12 m; distance between point E and point F: 12 m. The colored lines represent the trajectories of the first nine riders traced from point A, where the front wheels of the bikes cross the line between G and H, and point B, where they cross the line between E and F. We also reported the distance between Zarco’s Front Wheel and point G (FWG) and between his Front Wheel and point E (FWE): FWG: 0.98 m; FWE: 3.90 m.

The first video was provided by a fixed camera near the track, while the other two videos were from the onboard cameras of Rins (second video) and Rossi (third video). In all three videos, but from different angles, we identified point A as the exact instant when the front wheel of Zarco’s bike crossed the line between points G and H (Figure 4), where a shadow was cast directly under the ‘myWorld’ billboard, while point B indicates the exact instant when the front wheel of Zarco’s bike crossed the line formed between the first white stripe, clearly visible on the left side of the track (point E) and the point on the opposite side of the racetrack (point F, Figure 4), just before Zarco and Morbidelli’s bikes first made contact. Point C indicates the moment just before Morbidelli’s bike began crossing turn 3, while point D indicates the moment when it had completed its trajectory across turn 3 (Figure 5). In the first video, provided by the fixed camera positioned near turn 3, we can see the straightaway where the accident took place and turn 3 as Rossi and Viñales encountered the careening bikes. In order to measure the movement of each rider towards the middle of the track immediately after turn 2, we used the first video to calculate the distance that the first nine riders maintained between their Front Wheel and the inner edge of the track at point G (FWG) and point E (FWE) (Figure 6); hence, we calculated the delta between the two distances (Δ = FWE − FWG). In addition, we traced the trajectories of the first nine riders and reported the movement of Morbidelli and Zarco immediately before, during, and after Zarco’s overtaking of Morbidelli. In order to calculate the average speed of the bikes at various stages of the accident, we measured how long it took Zarco’s bike to travel from point A to point B and Morbidelli’s bike to travel from point B to point C and from point C to point D. Furthermore, examining the first video, we identified and analyzed the frame that captures the exact moment when Rossi encountered Morbidelli’s bike in turn 3. Considering the measurement of the diameter of Morbidelli’s rear wheel, we were able to measure how close the bike came to striking Rossi.

We also analyzed the second video from the onboard camera of Rins, who, at that moment in the race, was right behind Zarco and Morbidelli. The analysis of this footage allowed us to measure the average speed of Zarco and Rins before they headed into turn 2 and to identify the moment when the riders passed point A0 (corresponding to the point where the asphalt ends and the grass begins on the outer edge of the track; see Figure 3). Using Google Earth Pro, we then measured the distance between points A0 and A, and, using Kinovea, we measured the time it took Zarco and Rins to travel between those points. Moreover, in order to confirm the previous measurements made using the first video, the same calculations for Zarco and Morbidelli’s bikes were performed from points A to B and from points C to D. Using the third video provided by the onboard camera of Rossi, which shows the speed of the rider in real time, we tested the reliability of the indirect measurements. Specifically, we compared Rossi’s average speed from points A0 to A and from points A to B, calculated using both the average speed from all the collected frames and the time it took Rossi to travel the distances measured. To understand why Morbidelli’s bike did not strike Rossi, we analyzed Rossi’s actual speed and the lean angles from point A to the exact moment when Rossi encountered Morbidelli’s bike. Finally, we hypothesized two alternative scenarios and calculated the potential impact force of a collision between Rossi and Morbidelli’s bike.

## 3. Results

The distances between the key points, which were identified on the track and measured using Google Earth Pro, are reported in Table 1 (see Figure 3, Figure 4 and Figure 5), whereas the FWG and FWE distances of the first nine riders and their delta are reported in Table 2.

**Figure 7:**
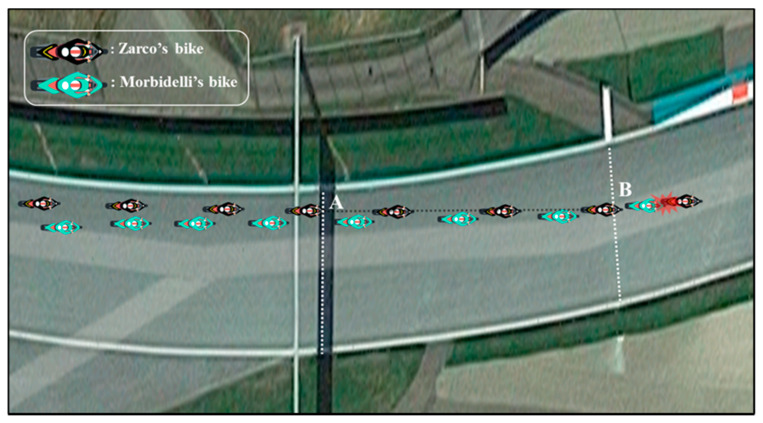
Representation of the dynamics of the accident showing that Zarco crossed Morbidelli’s trajectory immediately after overtaking him as he moved towards the center of the track.

From a qualitative standpoint, the analysis of the trajectory showed that Zarco, as he moved past Morbidelli, crossed the latter rider’s trajectory, moving 2.92 m towards the center of the track (see Table 2 and Figure 6 and Figure 7).

**Figure 8:**
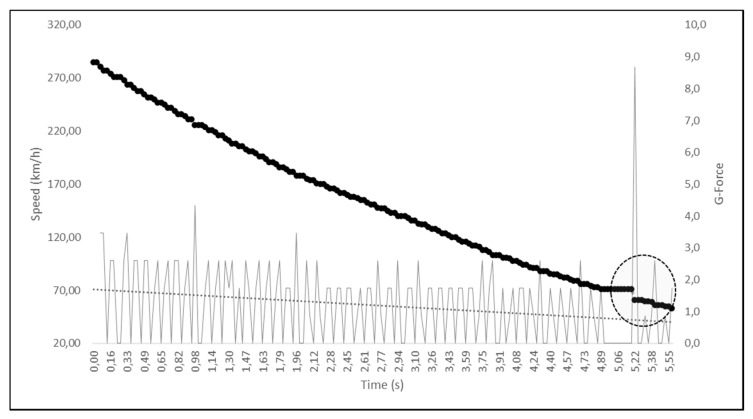
Rossi’s actual speed and instantaneous G-Force peak from point A to the point where he narrowly missed colliding with Morbidelli’s bike. The circled area shows the moment in which Rossi braked, reducing his speed by 10 km/h in 0.03 s.

Zarco’s movement towards the middle of the track was found to be greater than that of the other riders, whose movement was analyzed. Regarding the measurements of the time the motorbikes took to travel between the various points in the three different videos, we obtained the following results: based on footage from the first video, Zarco’s bike took 0.60 s to travel from point A to point B (44.5 m), at an average speed of 267 ± 7.9 km/h; Morbidelli’s bike took 3.93 s to go from point B to point C (205 m), at an average speed of 188 ± 1 km/h, and 0.66 s to travel from point C to point D (14 m), at an average speed of 76 ± 2.3 km/h. Analyzing the second video from Rins’ onboard camera, we determined that Zarco covered the 288 m from point A0 to point A in 3.43 s, at an average speed of 302 ± 1.8 km/h, whereas Rins covered the same distance in 3.46 s, travelling at an average speed of 299 ± 1.7 km/h. Moreover, from the same footage, we were able to determine that it took Morbidelli’s bike 0.60 s to cover the stretch from point A to point B, 3.93 s from point B to point C, and 0.66 s from point C to point D (confirming the previous measurements). Using the footage from Rossi’s onboard camera, we determined that it took Rossi’s bike 3.50 s to cover the stretch from point A0 to point A, at a calculated average speed of 296 ± 1.7 km/h, which corresponds to the average speed determined from the actual speeds recorded in the 105 frames analyzed from point A0 to point A (295.6 km/h). Furthermore, we found that Rossi’s actual average speed from point A to point B was 266 km/h, and it took him 0.60 s to cover the stretch from point A to point B, with an average indirectly calculated speed of 267 ± 7.9 km/h. These data confirm the reliability of our measurements and hence the quality of the analyses that were carried out based on those measurements. Analyzing Rossi’s real speed (exactly 172 frames) from point A to the moment in which he encountered Morbidelli’s bike (turn 3), we identified a point in which Rossi appeared to brake harder, as he entered turn 3, reducing his speed by 10 km/hr in 0.03 s, just 0.40 s before encountering Morbidelli’s bike (see Figure 8). 

**Figure 9:**
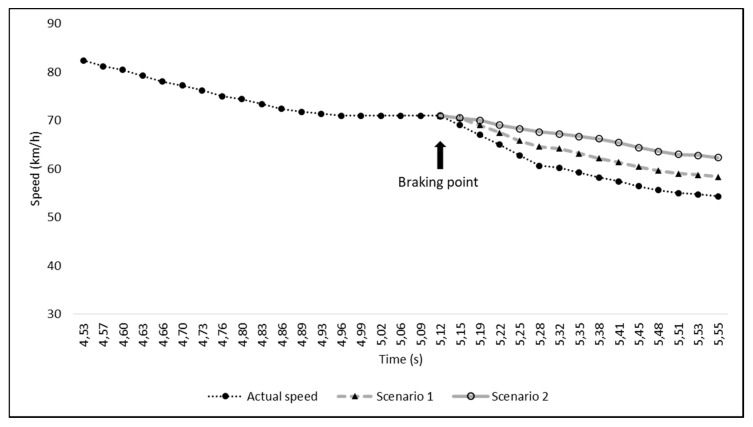
Rossi’s actual speed (moving an average of 5 points), 33 frames before the point where he narrowly missed colliding with Morbidelli’s careening bike. As described in the legend, the solid dots represent Rossi’s real speed and the solid triangles represent the speed in hypothetical scenario 1, while the empty dots represent the speed in scenario 2.

However, a deceleration of this magnitude is highly unlikely because, even if it were just for an instant, it would reach about 9 G. The braking system manufacturer, Brembo, claims that the “average” deceleration in the approach to turn 3 at the 2019 Austrian MotoGP was 1.23 G, and this value is in line with that which was analyzed here (from 285 to 53 km/h in 5.58 s, corresponding to an average of 1.2 ± 1.2 G). However, deceleration peaks are possible because rapid deceleration may occur with both sudden downshifting and with variations in the pressure applied by the rider to the brake lever. Indeed, analyzing the frame-by-frame G forces from Rossi’s deceleration curve (see Figure 8), we obtained instantaneous peaks that were even higher than 3.4 G. Hence, we proceeded to correct a possible data transmission error and/or lag by applying a moving average of five frames to reduce the G-force peak. Rossi’s deceleration thus appears more plausible. Indeed, as he rode through turn 3, he probably applied additional pressure to his brake lever, reducing his speed by 16.7 km/h in the 0.42 s before he encountered Morbidelli’s bike. We then developed two different hypothetical braking scenarios 15 frames before Morbidelli’s bike crossed Rossi’s path: in scenario 1, we hypothesized what would have occurred if Rossi had reduced his speed by 12.7 km/h, while in scenario 2, we postulated what would have occurred if he had reduced his speed by 8.7 km/h (see Figure 9).

**Figure 10:**
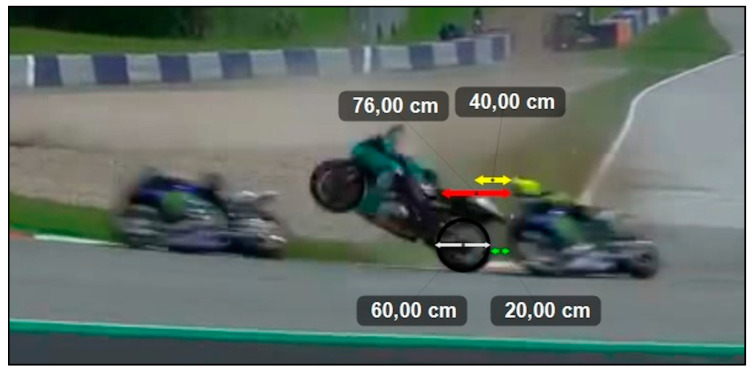
The frame capturing the exact instant when Morbidelli’s bike crossed Rossi’s path. Kinovea was used to measure the distances based on the measurement of the rear wheel of Morbidelli’s bike. Specifically, the white arrows indicate the 60-cm diameter of Morbidelli’s rear wheel (black circle); the green arrows represent the exact distance between Rossi’s front wheel and Morbidelli’s rear wheel; the yellow and red arrows indicate the distance that Rossi’s bike would have covered if he had slowed down according to scenario 1 (about 40 cm) and scenario 2 (about 76 cm), respectively.

Based on the calculation of the distance that would have been covered in the first hypothetical scenario, Rossi would have been 40 cm ahead of where he actually was on the track, while in the case of the second scenario, he would have been 76 cm ahead (see Figure 10). In addition, from the analysis of the frame in which Rossi encountered Morbidelli’s bike, we determined that the bike missed Rossi by just 20 cm. We also represented the points in which Morbidelli’s bike would have struck Rossi in the two hypothetical scenarios (see Figure 10).

Finally, based on footage from the first two videos, we can estimate with a good degree of accuracy that the speed of Morbidelli’s bike as it tumbled across turn 3 was about 76 km/h or 21.1 m/s. If it had struck Rossi at that speed, the consequences would certainly have been catastrophic. To have a more precise idea of those possible consequences, we would need to know the deceleration time of the bike following its impact with the rider. Fortunately, there was no impact, and thus, we were not able to calculate the deceleration time. We therefore calculated the force of the impact by hypothesizing that if the motorbike (150-kg) impacted an air-fence (soft-wall safety barrier) and we were to see a 2-m deformation of the barrier, we could calculate the deceleration time in the following manner Equation (1):

Deceleration Time (impact duration) = (2 × 2 m)/(21.1 m/s) = 0.19 s(1)

Hence, we would obtain the impact force (IF) Equation (2):

IF = (150 kg × 21.1 m/s)/(0.19 s) = 16.657 kN = 1698 kg(2)

The formula can easily be extended to calculate the approximate maximum impact force (peak impact force) by multiplying the resulting average impact force by two: 33.314 kN = 3396 kg.

## 4. Discussion

In the present report, using low-cost instruments, we performed a quali-quantitative analysis of the dynamics of the accident involving Zarco, Morbidelli, Rossi, and Viñales during the 2020 Austrian MotoGP. To our knowledge, this is the first time that a video analysis has been applied to an accident in a motorcycle race, whereas analyses of this kind are common for ordinary traffic accidents [12,13]. From our analyses, we were able to calculate that, in the straightaway leading into turn 2 (from A0 to A), Zarco was travelling at a higher average speed (302 ± 1.8 km/h) than either Rins or Rossi (299 ± 1.7 and 296 ± 1.7 km/h, respectively), probably because he was drafting off Morbidelli as he overtook him. In addition, from the average speeds of Zarco and Rossi in the stretch from point A to point B (266 km/h), we can conclude that the accident occurred as the riders were slowing down. Our analysis of the trajectories showed that, after point B, all the riders tended to move towards the middle of the track because of the layout of the circuit. However, the movement of Zarco’s bike (2.92 m) was greater than that of all the other riders’ bikes that were taken into consideration. Moreover, if we trace the trajectory of Zarco and Morbidelli (Figure 6 and Figure 7), we clearly see that, as he overtook Morbidelli, Zarco crossed his trajectory as he proceeded towards the middle of the track, just as Morbidelli did after having been overtaken, with his front wheel extremely close to Zarco’s rear wheel. It seems clear that Zarco made a risky maneuver choosing to pass Morbidelli at that point in the track and then moving towards the center of the track, knowing that the other rider was exceedingly close. Based on these analyses, it would seem that the layout of the track contributed to the scenario that played out, leading to the accident, and therefore, possible changes in the circuit might be considered. However, few data are available on the relative safety of motor racing circuit layouts. In fact, to the best of our knowledge, there is only one study [14] that analyzed whether changing the configuration of a motor racing circuit makes it safer. This particular study showed that the introduction of two bends into the fastest part of the track (similar to the one analyzed here) led to a decrease in the seriousness of auto racing accidents; however, these changes in the track seem to have resulted in a tendency for motorcycle racers to suffer slightly more serious injuries [14]. Hence, the decisions riders make to undertake or not to undertake certain maneuvers remain a key determining factor. Regarding the frame by frame analysis of Rossi’s speed as he entered and then rounded turn 3, it is very interesting to note his sudden deceleration, which, from a qualitative analysis of the images, appears to have been the result of his drawing too close to Vinales. If this was indeed the case, the presence of Vinales ahead of Rossi may have saved Rossi’s life or, in any case, led to his narrow avoidance of the collision. Indeed, if Rossi had braked slightly less, as hypothesized in scenarios 1 and 2, he would have been struck directly by Morbidelli’s bike. Finally, based on our analysis of the first and second video and the distance measured between points C and D, we calculated that Morbidelli’s bike travelled across curve 3 at a speed of 76 ± 2.3 km/h. Considering that a MotoGP bike weighs 150 kg, we hypothesized what the impact of the bike would be at that speed against air-fence barriers, obtaining a peak impact force of 33.3 kN. The tolerance of the human body to kinetic forces released in ordinary road accidents is limited. Injury is broadly related to the amount of kinetic energy applied to the human frame [15]. For example, a study estimated that the tolerance of the whole human neck to injury in tensile loading is as low as 3.1 kN for subjects with no pre-impact awareness and as high as 3.7 kN for subjects with a tensed cervical spine resulting from sufficient pre-impact awareness [16]. Considering that the peak impact force that we calculated was nine times higher than the maximum force tolerated by the human neck, if Morbidelli’s bike had struck Rossi at neck level, he would have had little chance of surviving the impact. On the other hand, if this impact had been absorbed by his entire body, we estimate that the impact force would have been comparable to the impact of a 65 kg (V. Rossi’s weight) body falling from a height of about 15 m (the fourth floor of a building). Although it is impossible to make further hypotheses regarding the possible consequences of the impact of Morbidelli’s bike against Rossi because of the countless unpredictable variables in play, our calculations help us to imagine the severity of such an impact.

### Limits and Strengths of the Study

Although the video frame rate limited precision to 0.03 s, we accounted for this error when calculating the speeds, showing that the upper and lower average error estimates were marginal and did not affect the quality of the analyses. In addition, the distances between the various points, though calculated precisely, taking into account the actual trajectories of the riders and their bikes, may contain some errors due to the different angles of the footage. Overall, our analysis allowed us to accurately describe the dynamics of the accident and its potentially catastrophic effects, while clearing up doubts regarding the speeds, distances, and trajectories of the riders who were involved.

## 5. Conclusions

This study, through a quali-quantitative analysis, helps us to gain a deeper understanding of the main causes and dynamics of the accident at the 2020 Austrian MotoGP Grand Prix, as well as the speeds of the motorcycles involved. Furthermore, we showed how V. Rossi narrowly avoided being struck by Morbidelli’s bike and what the consequences of other potentially tragic scenarios would have been if Rossi had braked differently or if he simply had not had his teammate in front of him. In addition, we have shown that, in the Austrian circuit, overtaking on the outside of the track in the straightaway between turn 1 and turn 2 is very risky, in particular, if it is completed at the end of turn 2 or at the point we have identified as point A. Hence, in light of our findings, regarding the riskiness of such a maneuver, it may be advisable for riders to be particularly prudent when attempting such a maneuver or not to repeat the same or a similar trajectory in this section of the Red Bull Ring circuit to overtake another rider in future races. Overall, this method could be used in future investigations to improve safety conditions and to gain a better understanding of the particular dynamics of specific sport motorcycle accidents.

## Figures and Tables

**Table 1:** Distances between key points on the racetrack.

From Point	To point	Distance (m)
A0	A	288
A	B	44.5
B	C	205.3
C	D	14
G	H	12
E	F	12

A0 = point where the asphalt ends and the grass begins on the outer edge of the track; A = point where the front wheels of the riders cross the line between points G and H; B = point where the front wheels of the riders cross the line between points E and F; C = point where Morbidelli’s bike began to cross the track of turn 3; D = point where Morbidelli’s bike ended to cross turn 3; G and H = the two ends of the line cast directly under the “myWorld” billboard; E and F = the two ends of the segment crossing the racetrack starting from the first white stripe on the left side of the track.

**Table 2:** Distances between the front wheels of the first nine riders and points G (FWG) and E (FWE) and their Delta. Zarco’s values are in bold.

Pilots	FWG (m)	FWE (m)	Δ (FEW − FWG) (m)
Espargaró	0.84	1.68	0.84
Dovizioso	0.3	1.65	1.35
Miller	1.35	3.6	2.25
Mir	0.39	1.5	1.11
Oliveira	0.28	1.81	1.53
Viñales	1.92	3.3	1.38
Rossi	0.83	2.5	1.67
Zarco	**0.98**	**3.9**	**2.92**
Rins	0.89	1.96	1.07

FWG = distance that the first nine riders maintained between their front wheel and the inner edge of the track at point G; FWE = distance that the first nine riders maintained between their front wheel and the inner edge of the track at point E.

## Supplementary Materials

The following are available online at https://www.mdpi.com/1660-4601/17/21/7989/s1, Video S1: AUSGP2020.
